# A Multirelational Social Network Analysis of an Online Health Community for Smoking Cessation

**DOI:** 10.2196/jmir.5985

**Published:** 2016-08-25

**Authors:** Kang Zhao, Xi Wang, Sarah Cha, Amy M Cohn, George D Papandonatos, Michael S Amato, Jennifer L Pearson, Amanda L Graham

**Affiliations:** ^1^ Department of Management Sciences Tippie College of Business The University of Iowa Iowa City, IA United States; ^2^ Interdisciplinary Graduate Program in Informatics The University of Iowa Iowa City, IA United States; ^3^ Schroeder Institute for Tobacco Research and Policy Studies Truth Initiative Washington, DC United States; ^4^ Department of Oncology Georgetown University Medical Center / Cancer Prevention and Control Program Lombardi Comprehensive Cancer Center Washington, DC United States; ^5^ Center for Statistical Sciences Brown University Providence, RI United States; ^6^ Department of Health, Behavior, and Society Bloomberg School of Public Health Johns Hopkins University Baltimore, MD United States

**Keywords:** social networks, smoking cessation, community networks

## Abstract

**Background:**

Online health communities (OHCs) provide a convenient and commonly used way for people to connect around shared health experiences, exchange information, and receive social support. Users often interact with peers via multiple communication methods, forming a multirelational social network. Use of OHCs is common among smokers, but to date, there have been no studies on users’ online interactions via different means of online communications and how such interactions are related to smoking cessation. Such information can be retrieved in multirelational social networks and could be useful in the design and management of OHCs.

**Objective:**

To examine the social network structure of an OHC for smoking cessation using a multirelational approach, and to explore links between subnetwork position (ie, centrality) and smoking abstinence.

**Methods:**

We used NetworkX to construct 4 subnetworks based on users’ interactions via blogs, group discussions, message boards, and private messages. We illustrated topological properties of each subnetwork, including its degree distribution, density, and connectedness, and compared similarities among these subnetworks by correlating node centrality and measuring edge overlap. We also investigated coevolution dynamics of this multirelational network by analyzing tie formation sequences across subnetworks. In a subset of users who participated in a randomized, smoking cessation treatment trial, we conducted user profiling based on users’ centralities in the 4 subnetworks and identified user groups using clustering techniques. We further examined 30-day smoking abstinence at 3 months postenrollment in relation to users’ centralities in the 4 subnetworks.

**Results:**

The 4 subnetworks have different topological characteristics, with message board having the most nodes (36,536) and group discussion having the highest network density (4.35×10^−3^). Blog and message board subnetworks had the most similar structures with an in-degree correlation of .45, out-degree correlation of .55, and Jaccard coefficient of .23 for edge overlap. A new tie in the group discussion subnetwork had the lowest probability of triggering subsequent ties among the same two users in other subnetworks: 6.33% (54,142/855,893) for 2-tie sequences and 2.13% (18,207/855,893) for 3-tie sequences. Users’ centralities varied across the 4 subnetworks. Among a subset of users enrolled in a randomized trial, those with higher centralities across subnetworks generally had higher abstinence rates, although high centrality in the group discussion subnetwork was not associated with higher abstinence rates.

**Conclusions:**

A multirelational approach revealed insights that could not be obtained by analyzing the aggregated network alone, such as the ineffectiveness of group discussions in triggering social ties of other types, the advantage of blogs, message boards, and private messages in leading to subsequent social ties of other types, and the weak connection between one’s centrality in the group discussion subnetwork and smoking abstinence. These insights have implications for the design and management of online social networks for smoking cessation.

## Introduction

Over the past decade, many people have turned to the Internet to find health-related information and support. According to the Pew Research Center, 72% of adult Internet users in the United States use the Internet for health-related purposes. Of those, 26% have read or watched someone else’s experience about health or medical issues in the last 12 months and 16% have used the Internet to find others who might share the same health concerns in the last year [1]. Interactions with peers who share similar health problems are facilitated by online health communities (OHCs), which are Internet-based online groups or websites specifically designed for both patients and caregivers to learn about an illness, seek and offer support, and connect with others in similar circumstances [2]. Online health communities enable individuals to connect via forums, discussion boards, private messages, and other forms of synchronous and asynchronous social interaction. In addition to their popularity, the physical and psychological benefits of participation in OHCs have been well documented in numerous studies (eg, [3-6]). Given the proliferation and popularity of OHCs, it is important to understand the experiences and behaviors of users in these network contexts so that the design and management of OHCs can be improved or optimized.

Various aspects of OHCs have been studied, such as topics of online discussions [7-11], the nature and exchange of various types of social support [12-15], users’ participation patterns [16-19], and the psychological mechanisms through which participation affects health outcomes [20-22]. Less studied have been the social network structures of OHCs and the role of network characteristics in understanding individual user patterns and outcomes. Social network analyses can help identify community structures at the network level (ie, considering the entire network), as well as individual behaviors and positions at the individual level (ie, considering individuals and their ties with peers). To date, social network analyses of OHCs have largely focused on social networks based on a single type of communication (eg, posting comments to threaded discussions [23,24]) or have aggregated different types of communications into one network [25]. However, most social networks—both online and offline—are multirelational (also called multiplex or multidimensional networks), composed of myriad social relationships with family members, neighbors, classmates, colleagues, etc [26]. In OHCs, users' communications via different channels foster different types of social relations or ties. For example, private message ties may be more intimate and influential than ties formed based on the exchange of messages in a group discussion. Multirelational analyses of social networks have provided important new insights into information flows, individual centralities, growth models, link prediction, and community discoveries. For example, social ties based on one type of relationship can predict the formation of ties based on another type of relationship [26]. Differentiating social ties based on different relationships can also contribute to the prediction of individuals' preferences [27,28].

This study adopted a multirelational perspective in examining the network structure and dynamics of a popular OHC for smoking cessation. We examined the structure of the social network, as well as the coevolution of different types of subnetworks. Numerous publications highlight the importance of social influences on a range of smoking behaviors, including initiation, cessation, and relapse, in offline settings. Thus, we also illustrated how users’ behavior patterns in different subnetworks were related to their smoking status, using outcome data available for a subset of OHC members enrolled in a randomized trial. Our primary goals were to characterize multirelational social networks in an OHC for smoking cessation, identify dynamic coevolution of multirelational networks, and explore potential links between users’ online social network engagement and health behavior using a multirelational approach. To our knowledge, this study is the first to analyze large-scale multirelational social networks among OHC users of a Web-based, smoking cessation program. Furthermore, while previous studies have enumerated social networks based only on users’ posting behaviors, our multirelational social network incorporated private behaviors as well by considering both posting and reading behaviors of users. This study lays the foundation for an ongoing series of analyses aimed at understanding and optimizing the multirelational behaviors of a large OHC for smoking cessation.

## Methods

### Intervention

We conducted these analyses using longitudinal data from BecomeAnEX, a Web-based smoking cessation program developed and managed by Truth Initiative (formerly American Legacy Foundation). Launched in 2008, BecomeAnEX was developed in accordance with the Clinical Practice Guidelines for Treating Tobacco Use and Dependence [29]. Through an interactive, multimedia experience, BecomeAnEX assists users in setting a quit date, understanding their smoking habits and preparing to quit, selecting and using Food and Drug Administration–approved medications, and connecting with others for social support in the BecomeAnEX community. A national mass media campaign [30] and ongoing Web-based advertising have resulted in more than 700,000 registered users since its inception.

The BecomeAnEX community is composed of thousands of current and former smokers who interact via 4 primary communication channels. Users can exchange private messages via the site; users who have opted-in to receive email notifications are informed when they have received a new message. Message board posts are public communications made on a member’s profile page. All users have a community profile that can be customized with photos and personal information. Group discussions are threaded discussions among users with similar experiences or interests (eg, “March Quit Dates,” “Over 50 BecomeAnEXs”). Blogs are single entries made by users about their experiences, which appear in reverse chronological order on the site. Users can comment on others’ blog posts, creating threaded discussions similar to group discussions. Communication between and communication among members via blogs (and comments), message boards, and group discussions are all public communications that can be accessed by all BecomeAnEX users. Private messages occur only between two users. Blogs and group discussions elicit many-to-many communications, whereas posts on message boards and private messages are one-to-one communications. A community administrator addresses technical issues and spammers, but otherwise the community is largely unmoderated.

All user actions are date and time stamped and stored in a relational database. Before analysis, users’ identifiers were converted into alphanumeric strings using cryptographic hash functions, which makes this conversion infeasible to invert. The content of private messages was not included in the dataset to protect privacy.

### Multirelational Social Network Analyses

The Python programming package NetworkX (v1.11) was used to construct and analyze social networks. The multirelational network consists of 4 subnetworks: private messages (PM), message boards (MB), group discussions (GD), and blogs (BL). In each subnetwork, a node represents an individual user, while a directed tie pointing from user A to user B means that B accessed information contributed by A or, in other words, information from A reached B. Taking the blog subnetwork as an example, if B posted a comment to one of A’s blogs then we assume B read (or at least skimmed) the original blog post, and so there is a tie pointing from A to B (A→B) indicating that A’s contribution has reached B. Similarly, if A’s clickstream (ie, the logs of clicking URLs) suggests that he or she has read that comment from B, then we add a B→A tie to reciprocate the earlier A→B tie. In such a directed network, a node’s in-degree refers to the number of other nodes that have ties pointing to it (ie, the number of people who may have influenced that user). Conversely, a node’s out-degree is the number of its outgoing ties (ie, the number of people that user has potentially influenced). A node’s total degree is the total number of its network neighbors irrespective of tie direction. By incorporating both posting (outgoing ties if a post was read by others) and reading (incoming ties) behaviors, our subnetworks can better capture how information flows among OHC users via each means of communication. When combining all nodes and ties in the 4 subnetworks, an aggregated network emerges, where a tie means two users have had some type of interaction in the community.

Our analysis proceeded in 4 steps. First, we conducted topological analysis to illustrate the characteristics of the 4 subnetworks. We examined the number of nodes with total degree greater than zero, the number of edges, density (defined as the number of actual ties divided by the number of possible ties), and the proportion of ties that were reciprocated. To compare the connectedness of the subnetworks, we identified the largest strongly connected component (LSCC). A strongly connected component is a subset of a network, in which there is a directed path between every pair of nodes. The LSCC is the one with the most nodes among all strongly connected components of a network. For each subnetwork, we also calculated the average shortest path among nodes in its LSCC. In general, the larger the LSCC and the shorter the average path length within the LSCC, the more connected the network.

Second, we measured structural similarities among the subnetworks using 2 metrics: centrality correlations at the individual level and tie overlap at the network level. At the individual level, one’s centrality can be captured by in- and out-degrees. Higher degrees usually mean higher centralities. We correlated each node’s rank by in- and out-degrees in one subnetwork with the same node’s rank by in- and out-degrees in the other 3 subnetworks. A high correlation coefficient between two subnetworks suggests that individuals with high centrality in one subnetwork tend to have high centrality in another. At the network level, the tie overlap between two subnetworks was calculated with Jaccard coefficients [31]. A high Jaccard coefficient between two subnetworks signals that if there is a tie from node *i* to node *j* in one subnetwork, there is a high probability that a tie also exists from *i* to *j* in another subnetwork.

Third, coevolution analysis was used to demonstrate tie formation dynamics across subnetworks. Building on analyses of the static characteristics (ie, topology) and structural similarities of the subnetworks, we also investigated coevolution dynamics between the 4 subnetworks. We were specifically interested in how the formation of a tie between two users in one subnetwork triggered the formation of ties between the same two users in other subnetworks. For each subnetwork, we calculated the probability that this subnetwork hosts the first tie among all pairs of nodes that were connected in any of the 4 subnetworks. We also investigated whether the same pair of nodes that formed their first tie in one of the subnetworks would form new ties in other subnetworks. To answer this question, we analyzed the temporal sequence of tie formations, and calculated the probabilities to form subsequent ties in the second and third subnetworks given the subnetwork in which the first tie was formed, along with the most common tie sequences.

Finally, user profiling was used to identify whether centralities in different subnetworks had different implications for abstinence rates. We used Gaussian mixture models (GMMs), an unsupervised clustering technique, to divide users into groups based on their centralities in the 4 subnetworks so that those with similar centralities across subnetworks were placed in the same group. As the input for the profiling process, each user is represented by a vector with 8 elements, each one being the user’s in- and out-degree in the 4 subnetworks. To determine the number of user groups (K), we tried different K values (from 2 to 10) for GMM and selected the value that represented the best fit with our data as determined by log-likelihood.

The user profiling analysis was based on a subsample of N=1337 BecomeAnEX users who participated in a randomized smoking cessation trial (NCT01544153) and were assigned to the control arm (BecomeAnEX alone). The trial has been described in detail elsewhere [32]. All participants were current smokers at baseline; 30-day point prevalence abstinence was assessed at 3 months after enrollment (“In the past 30 days, have you smoked any cigarettes at all, even a puff?”). The overall response rate for the trial at 3 months was 58.41% (781/1337). Users who did not complete the follow-up survey were conservatively counted as smoking under the intent-to-treat principle. Of the 1337 BecomeAnEX users in this sample, 12.27% (164/1337) reported 30-day point prevalence abstinence at 3 months. Differences in abstinence rates between the user groups identified in the GMMs described above were examined using analysis of variance.

The study protocol was approved by Chesapeake Institutional Review Board (protocol #CR00040526).

## Results

### Description of Dataset

The dataset used in this study spanned the period from January 1, 2010, to May 31, 2015, and included records of both posting and reading behaviors of N=71,251 users who accessed content of the community on BecomeAnEX by clicking and reading a post (eg, a blog, a message board post, or a group discussion thread) or a private message. The community was migrated from a different platform before this period, which resulted in a slightly different user experience. Our analyses focus on this time frame given the stability of the social network feature set.

### Topological Analysis

**Figure 1:**
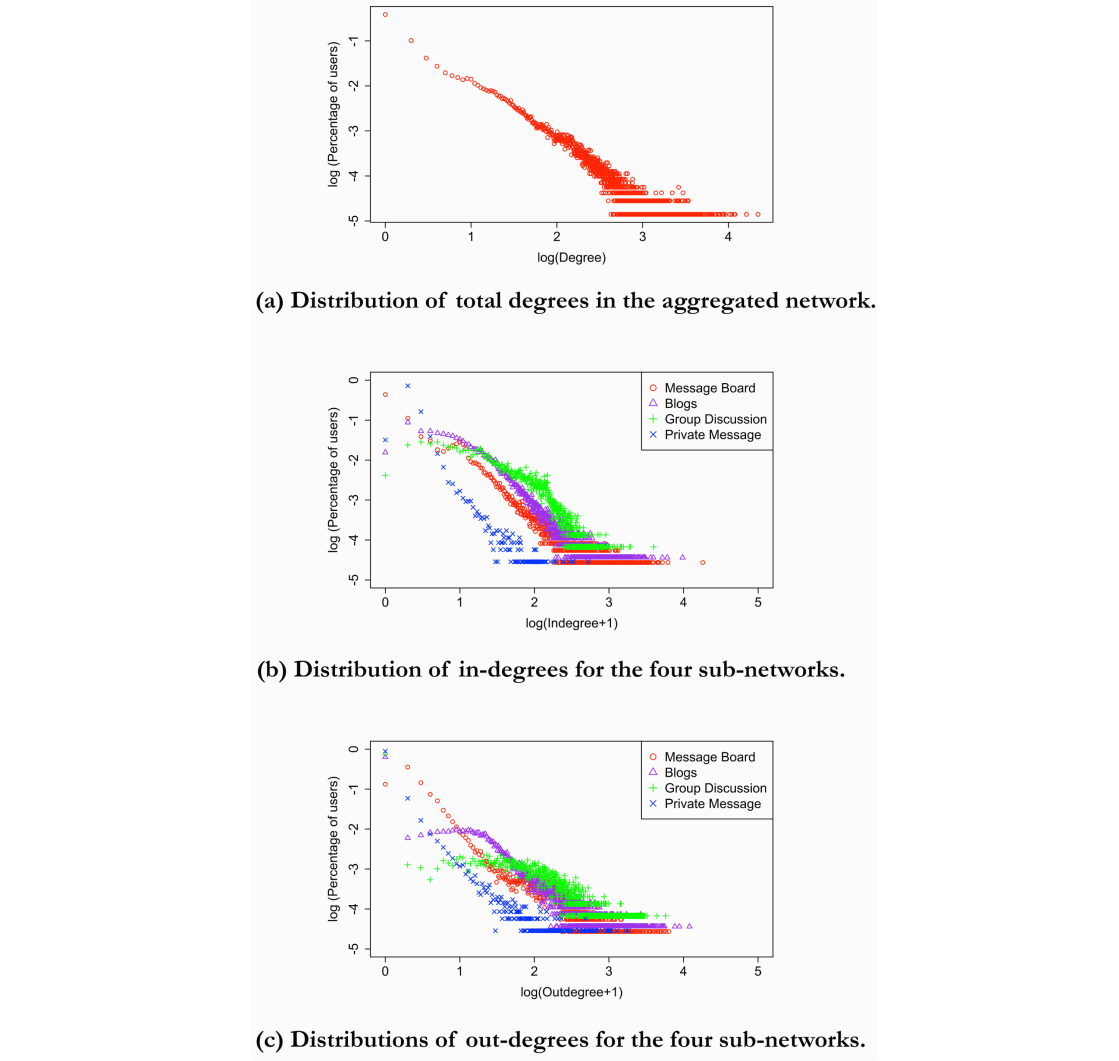
Network degree distributions for the aggregated network and 4 subnetworks.

Figure 1 shows distributions of total degrees in the aggregated network (part A), in-degrees (part B), and out-degrees (part C) for the 4 subnetworks. The distribution of total degrees in the aggregated network was similar to the power-law degree distribution that is typical for a scale-free network. In a power-law degree distribution, the probability that a node has degree *k* follows *P(k)=c* × *k*^−r^, where *c* and *r* are network-specific constants. In a log-log plot, a power-law degree distribution features a downward-sloping straight line that is similar to Figure 1, part A. However, the in- and out-degree distributions of the 4 subnetworks suggested that each subnetwork, in fact, had different topological characteristics. The private message subnetwork featured power-law distributions for both in- and out-degrees, but the other 3 were hardly scale-free networks as their curves in log-log plots were nonlinear. For example, the blog and group discussion subnetworks had relatively flat distributions for low in- and out-degrees. On the one hand, blog and group discussion distributions conformed to the generally observed pattern among scale-free networks that nodes with higher degrees appear less frequently. On the other hand, the message board and the group discussion subnetworks featured sudden increases in the number of nodes with in-degree around 10 and 18, respectively. Additionally, there were more users with zero out-degrees than those with zero in-degrees, because many users only read community content without contributing and thus had no outgoing ties.

Descriptive statistics of the aggregated network and each of the 4 subnetworks are presented in Table 1. Among the 4 subnetworks, the message board subnetwork had the most nodes, followed by the private message subnetwork. However, the private message subnetwork also had the lowest density. The high number of nodes with nonzero degree in the private message subnetwork was attributable to many nodes with in-degree of 1. The presence of welcome messages was also reflected by the low reciprocity of the private message subnetwork: only 8.94% (4970/55,585) of the ties were reciprocated. By contrast, even though its number of nodes ranked only third among the 4, the blog subnetwork had the shortest average path length in the LSCC, the second highest density, and the second highest reciprocity rate, indicating a well-connected network in which people actively interacted with each other. Among the 4 subnetworks, the private message subnetwork was the least connected with the smallest LSCC (6.87% (2404/34,996) of nodes in the LSCC) and the longest average shortest path in the LSCC (3.74).

### Structural Similarity

The topological analysis described in the previous section treated each subnetwork as independent. However, two individuals may be connected in more than one subnetwork in the online community. We computed how many pairs of nodes were connected in different subnetworks. As shown in Table 2, although many pairs of nodes were connected in only 1 subnetwork, there were still more than 370,000 pairs of nodes that were connected in 2 or more subnetworks.

As shown in Tables 3 and 4, the blog and message board subnetworks had the most similar topologies. They had the highest correlation in node centralities (ρ=.45), as well as the top Jaccard coefficient (.23) that was at least 4 times higher than the others. Meanwhile, the private message subnetwork was quite different from the other 3 subnetworks. Although those with high out-degree in the private message subnetwork also tended to have high out-degree in other subnetworks (with moderate correlations), in-degree in the private message subnetwork was negatively correlated with in-degrees in other subnetworks. Active contributors in the other 3 subnetworks tended to send messages to more people, but those who received messages from more people did not necessarily read more posts from others.

**Table 1:** Descriptive statistics of the aggregated network and the 4 subnetworks.

Characteristics	Aggregated	Blog	Message board	Group discussion	Private message
Number of nodes with degree >0	71,251	27,461	36,536	14,827	34,996
Number of edges	2,578,659	1,065,514	1,027,694	956,506	60,555
Density	5.08×10^−4^	1.41×10^−3^	7.70×10^−4^	4.35×10^−3^	4.94×10^−5^
% Of reciprocated ties	18.22 (397,339/2,181,320)	23.61 (203,485/862,029)	29.62 (234,873/792,821)	3.57 (32,928/923,578)	8.94 (4970/55,585)
% Of nodes in the LSCC^a^	35.64 (25,395/71,251)	35.00 (9611/27,461)	41.61 (15,203/36,536)	26.48 (3926/14,827)	6.87 (2404/34,996)
Average shortest path length in LSCC	2.86	2.29	2.68	2.40	3.74

^a^LSCC: largest strongly connected component.

**Table 2:** The number of node pairs with ties in different networks.

Node pairs	Number of node pairs
Pairs connected in 1 subnetwork only	1,807,720
Pairs connected in 2 subnetworks	300,758
Pairs connected in 3 subnetworks	66,591
Pairs connected in 4 subnetworks	6251

**Table 3:** Spearman rank correlation coefficients between individual nodes’ in-degree (above the diagonal) and out-degree (below the diagonal) across the 4 subnetworks.

Network	Blog	Message board	Group discussion	Private message
Blog	—	.45^a^	.35^a^	−.23^a^
Message board	.55^a^	—	.40^a^	−.10^a^
Group discussion	.33^a^	.32^a^	—	−.10^a^
Private message	.43^a^	.35^a^	.35^a^	—

^a^*P*<.001.

**Table 4:** Tie overlap measured by Jaccard coefficients between the 4 subnetworks.

Subnetwork	Blog	Message board	Group discussion	Private message
Blog	—	0.23	0.05	0.02
Message board	—	—	0.05	0.02
Group discussion	—	—	—	0.01

### The Coevolution of Multirelational Networks

As shown in Table 5, the largest proportion of first ties (39.24% (855,893/2,181,320)) occurred in the group discussion subnetwork, 33.67% (734,559/2,181,320) occurred in the blog subnetwork, 28.30% (617,287/2,181,320) occurred in the message board subnetwork, and only 1.87% (40,728/2,181,320) occurred in the private message subnetwork, which had the fewest edges. Among those who formed their first ties in the blog subnetwork, 27.22% (199,913/734,559) formed their second ties in another subnetwork, most commonly the message board subnetwork as BL→MB is the most frequent 2-tie sequence. Also, for pairs that were first connected in the blog subnetwork, only 4.37% (32,126/734,559) were eventually connected via a third tie, with BL→MB→GD being the most frequent 3-tie sequence. Comparing the conditional probabilities of forming the second tie given the first tie in each subnetwork, we found that a first tie in the blog and message board subnetworks had similar probabilities of leading to a second and third tie in other subnetworks. By contrast, first ties in the group discussion subnetwork had the lowest probability of developing into subsequent ties.

**Table 5:** Probabilities (P) of subnetworks to host the first tie between two nodes, conditional probabilities of subsequent ties in other subnetworks, and top tie sequences.

Subnetwork hosting the 1st tie	P(hosting the 1st tie), %	P(forming 2nd ties in other subnetwork | 1st tie), %	Top 2-tie sequence by P(sequence | 1st tie), %	P(forming 3rd ties in other subnetwork | 1st tie), %	Top 3-tie sequence by P(sequence | 1st tie), %
Blog	33.67 (734,559/2,181,320)	27.22 (199,913/734,559)	BL^a^→MB^b^ 86.03 (135,245/157,212)	4.37 (32,126/734,559)	BL→MB→GD^c^ 9.23 (14,517/157,212)
Message board	28.30 (617,287/2,181,320)	28.52 (176,031/617,287)	MB→BL 79.52 (96,418/121,244)	5.10 (31,492/617,287)	MB→BL→GD 11.47 (13,909/121,244)
Group discussion	39.24 (855,893/2,181,320)	6.33 (54,142/855,893)	GD→MB 58.43 (27,795/45,573)	2.13 (18,207/855,893)	GD→BL→MB 14.47 (6,882/47,573)
Private message	1.87 (40,728/2,181,320)	26.05 (10,611/40,728)	PM^d^→BL 44.2 (378/855)	3.65 (1487/40,728)	PM→MB→BL 5.1 (44/855)

^a^BL: blog.

^b^MB: message board.

^c^GD: group discussion.

^d^PM: private message.

### User Profiling and Abstinence

Gaussian mixture model with K=7 generated user groups that fit our data the best—the log-likelihood reached a plateau when K=7. Adding more clusters only increased the likelihood by 0.4%-4% (K=8, 9, and 10), but lower K values (K=2 to 6) reduced the likelihood by 16%-61%. Table 6 lists the average centrality (in- and out-degrees in the 4 subnetworks) of each of the 7 groups, along with the number of users and 30-day point prevalence abstinence (ppa) rates at 3 months for each user group. Groups are sorted from the highest to the lowest abstinence rates.

**Table 6:** User groups and their average in- and out-degrees in 4 subnetworks.

User group	MB^a^ In	MB Out	BL^b^ In	BL Out	GD^c^ In	GD Out	PM^d^ In	PM Out	No. of users	30-Day ppa at 3 months^e^, %
1. Super users	118.8	150.1	176.9	183.7	118.9	30.2	6.3	6.3	18	55.6 (10/18)
2. Regular contributors	8.8	4.5	17.8	9.4	51.5	95.0	0.5	0.0	13	38.5 (5/13)
3. Regular contributors	11.1	19.1	24.8	25.8	17.8	0.0	0.7	0.3	88	30.7 (27/88)
4. Lurkers	3.9	1.0	12.6	0.0	56.4	0.0	0.4	0.0	68	14.7 (10/68)
5. Lurkers	3.0	0.7	14.4	0.0	0.0	0.0	0.4	0.0	118	14.4 (17/118)
6. Inactive users	0.0	0.9	0.0	0.0	0.0	0.0	1.2	0.0	210	9.5 (20/210)
7. Inactive users	0.0	0.0	0.0	0.0	0.0	0.0	0.0	0.0	822	9.1 (75/822)

^a^MB: message board.

^b^BL: blog.

^c^GD: group discussion.

^d^PM: private message.

^e^The 30-day point prevalence abstinence (ppa) at 3 months calculated under intent-to-treat principle with nonresponders counted as smokers.

**Figure 2:**
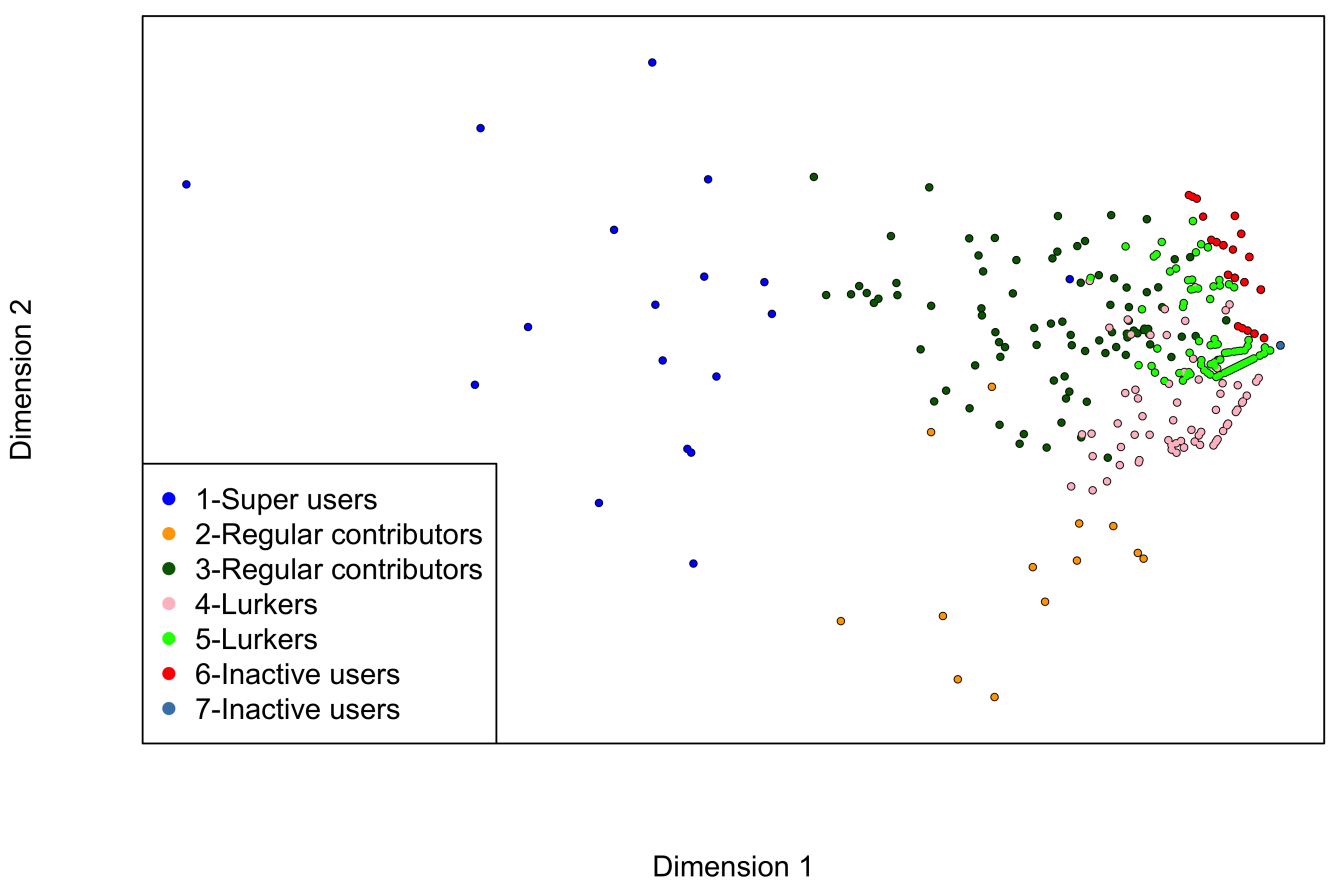
Multidimensional scaling of the 7 user groups.

Users in group 1 were highly connected users with many incoming and outgoing ties across the 4 subnetworks. Groups 2 and 3 represented regular contributors who not only read what others posted, but also contributed content that was read by others, although they were less connected than those in group 1. Groups 4 and 5 were “lurkers” who mainly read posts from others but contributed little or no content of their own. The largest 2 groups (groups 6 and 7) consisted of trial participants who never visited the BecomeAnEX community (but may have used other smoking cessation features or content on the website), although those in group 6 received private messages and visits to their message boards from an average of about 1 other user. Figure 2 shows the differences among the 7 groups of users after using multidimensional scaling to map the 8-dimensional data into 2-dimensional space.

The overall comparison between user groups found that high degree centralities were associated with high abstinence rates. For example, well-connected users in group 1 had significantly higher abstinence rates than regular contributors in group 3 (*F*_1,__104_=4.15, *P*=.04), lurkers in group 4 (*F*_1,__84_=15.38, *P*<.001) and group 5 (*F*_1,__134_=18.66, *P*<.001), and isolated users in group 6 (*F*_1,__226_=35.22, *P*<.001) and group 7 (*F*_1,__838_=43.83, *P*<.001). Regular contributors in groups 2 and 3 also had significantly higher abstinence rates than lurkers (group 2 vs group 4: *F*_1,__79_=4.19, *P*=.04; group 2 vs group 5: *F*_1,__129_=4.96, *P*=.03; group 3 vs group 4: *F*_1,__154_=5.53, *P*=.02; group 3 vs group 5: *F*_1,__204_=8.19, *P*=.005) and inactive users (group 2 vs group 6: *F*_1,__221_=10.70, *P*<.001; group 2 vs group 7: *F*_1,__833_=12.88, *P*<.001; group 3 vs group 6: *F*_1,__296_=22.32, *P*<.001; group 3 vs group 7: *F*_1,__908_=38.61, *P*<.001). The robustness of these findings is supported by additional analyses that examined abstinence rates under a less-conservative, responder-only approach. Abstinence rates for groups 1 to 7 were 71.4% (10/14), 55.6% (5/9), 39.1% (27/69), 24.4% (10/41), 21.5% (17/79), 16.4% (20/122), and 16.8% (75/447), respectively. The rank order of the 7 groups is largely consistent, with the exception of group 7, which has a slightly high abstinence rate than group 6 under this analytic approach.

The multirelational network approach enabled the discovery of meaningful subgroups of participants, using information that would have been lost in an aggregated network analysis. For example, users in group 3 and group 4 had similar total degrees in the aggregated network (73.8 and 71.7, respectively). However, Table 6 reveals that members of group 3 were active across all subnetworks, whereas members of group 4 were active almost exclusively in the group discussion subnetwork. These patterns were significantly associated with abstinence as we showed in the previous paragraph (*F*_1,__154_=5.53, *P*=.02), suggesting a weak relationship of centrality in the group discussion subnetwork with abstinence.

In addition, the specific subnetwork in which users gained their centralities resulted in varying abstinence rates. For instance, having high in- and out-degrees in the group discussion subnetwork alone did not necessarily suggest high abstinence rates. Lurkers in group 4 had the second highest average in-degree in the group discussion subnetwork, but the abstinence rate in group 4 was not significantly different from that of otherwise similarly connected lurkers in group 5 (*F*_1,__184_=0.003, *P*=.96), or of isolated users in group 6 (*F*_1,__276_=1.43, *P*=.23) or group 7 (*F*_1,__888_=2.27, *P*=.13). Similarly, group 2 had the highest average out-degree in the group discussion subnetwork, yet its abstinence rate was not significantly different from users in group 3 (*F*_1,__99_=0.31, *P*=.58), who had much lower centralities in the group discussion subnetwork but higher centralities in the blog and message board subnetworks.

## Discussion

### Principal Findings

To our knowledge, this study is the first to analyze a smoking cessation OHC from the perspective of a multirelational social network. We constructed 4 subnetworks based on users’ interactions via 4 communication channels and illustrated the value of a multirelational approach through topological analysis, coevolution analysis, and user profiling analysis. We found that the subnetworks based on different types of relationships had different topological characteristics. Specifically, the blog subnetwork was the most connected. The blog and message board subnetworks were topologically similar, whereas the private message subnetwork was topologically distinct from others.

Coevolution analyses of subnetwork tie formation dynamics found that although the group discussion subnetwork was the most common subnetwork for the initial formation of ties between users, ties formed there also had the lowest probability of leading to additional ties in another subnetwork. This may have been because the many-to-many group-based interactions did not encourage relationship building at the dyadic (ie, one-to-one) level. By contrast, roughly a quarter of users who formed their first ties in one of the other subnetworks, including in the private message subnetwork, went on to form additional ties in a second subnetwork. When two BecomeAnEX users are first connected via private messages, it is likely to be via a welcome message from one member to another. Even though many such messages may be a mere formality, they do seem to encourage users to build more ties in other social networks, notably the blog and the message board subnetworks. However, because we did not use the content of private messages to protect users’ privacy, we cannot directly validate whether these messages were indeed welcome messages.

User profiling based on users’ centralities across the 4 subnetworks showed that users can have different centralities in different subnetworks. This further highlights the importance of examining subnetworks within OHCs. For example, although users with high centralities across all 4 subnetworks had high abstinence rates, aggregating these subnetworks into one network would have lost valuable information about users’ online and offline behaviors. In other words, having high total degrees, high in-degrees, or high out-degrees in the aggregated network was not necessarily related to abstinence. Instead, our multirelational approach revealed that the subnetwork in which a user gained his or her centralities mattered.

Analyzing centrality with a multirelational approach is likely to be particularly useful for researchers and website designers interested in improving the effectiveness of OHCs as health interventions. This approach is capable of identifying which communication channels are facilitative of desired outcomes and which channels are not. We found that high centrality in the blog and message board subnetworks was positively associated with abstinence, whereas high centrality in the group discussion subnetwork was not. Recall that the group discussion subnetwork has the lowest reciprocity rate of 3.57% (32,928/923,578). Having a high degree in this subnetwork does not necessarily mean the user interacted or bonded with more peers in the community. These findings suggest that the group discussion feature may not be contributing to the health behavior change goals of the OHC and may be a candidate for revision to serve a more useful function or removal so as to avoid distracting new users from more active and/or effective communication channels. These insights would have been obscured with an aggregated network analysis; the multirelational approach allowed the signal of blog and message board centrality to be distinguished from the noise of group discussion centrality.

### Implications

These findings shed light on users’ online behaviors in a multirelational social network in an OHC for smoking cessation and inform community design or redesign, management, and interventions for smoking cessation and other health-risk behaviors using Web-based platforms for behavior change. For example, because the blog and the message board subnetworks were similar in structure and often triggered the formation of subsequent ties in each other, better integration of blogs and message boards may help users connect with each other more easily. Private messages can be a good way to welcome new users and encourage them to build more ties with peers using other means of communication, such as visiting message boards. Conversely, group discussions had the lowest probabilities of triggering subsequent ties in other subnetworks.

### Comparison With Prior Work

Our observation that users with higher centralities had higher abstinence rates is consistent with previous research on the role of online social networks in smoking cessation. Two recent studies [33,34] demonstrated that smokers who participated in an online community—even just browsing or “lurking” the posts made by others—were more likely to be abstinent than those who did not participate at all in the community. These studies used statistical methods to account for the possibility of selection bias (ie, more active users of an OHC may be more motivated to make changes to their behavior), lending credence to causal links between online community engagement and smoking outcomes. Given the observational nature of the analyses in this paper, however, we cannot conclude that social network position, per se, is causally related to abstinence. Nevertheless, understanding more about behaviors within a social network highlights factors associated with positive outcomes. These factors could be harnessed in future interventions to improve longer-term cessation rates. Other studies have also identified the existence of key established members who have different roles within a smoking cessation network [19,35,36], but these studies have primarily focused on user behaviors or content of posts and have yet to link these behaviors to abstinence outcomes.

Although previous social network research has adopted the multirelational approach to study online social networks, the focus was mainly on traditional network analysis tasks, such as node ranking, link prediction, network evolution, and community discoveries [25,26,29-31,37,38]. Few have explored individual behaviors in the context of multirelational social networks, especially offline behaviors.

### Limitations

This research has a few limitations. First, we showed that users with different roles based on their centralities in subnetworks can have different abstinence rates, but we cannot make causal statements regarding the links between centralities in certain subnetworks and abstinence. Second, the user profiling analysis was based only on a group of users who enrolled in a randomized trial. Third, we considered only the social network among users and did not incorporate the textual content of their interactions. This would be an interesting direction for future work to better understand what users shared and talked about in OHCs. Finally, we did not assess or examine other social influences that could affect smoking behaviors, such as family, friends, health care providers, and social media channels. It is important to determine whether and how these offline sources of social support interact with network dynamics that occur within OHCs for smoking cessation.

### Future Research Directions

Directions for future work include investigating how information flows between nodes via different channels of communication. Topic modeling techniques can be used to capture what people talked about in each communication channel to model and predict the coevolution of multirelational social networks. The outcome of topic modeling also has the potential to reveal the evolution of users into specific self-assigned roles within an online community (eg, “Elder,” “Conflict Resolver”). Future work with this network will seek to identify content, communication strategies, and network connections that improve abstinence outcomes.

### Conclusions

This study represents one of the first efforts to study the structure and dynamics of a large-scale OHC for smoking cessation. Specifically, user behavior patterns in the subnetworks were found to be differentially associated with important outcomes, including formation of subsequent ties to the network as well as abstinence from smoking. Whereas blogs, message boards, and private messages are effective in triggering subsequent social ties in other subnetworks, group discussions are not. Centralities in the group discussion subnetwork are not indicative of smoking outcome either. The results highlight the value of the multirelational approach in analyzing large-scale online social networks among OHC users. Our research also contributes to multirelational social network analysis by showing that multirelational network analysis of online ties can provide valuable insights for understanding individual health behaviors.

## Abbreviations

GMM: Gaussian mixture model
LSCC: largest strongly connected component
OHC: online health community
